# The professional relationship forms the base: Swedish child health care nurses’ experiences of encountering mothers exposed to intimate partner violence

**DOI:** 10.1080/17482631.2021.1988043

**Published:** 2021-10-25

**Authors:** Agneta Anderzén Carlsson, Charlotte Bäccman, Kjerstin Almqvist

**Affiliations:** a University Health Care Research Center, Faculty of Medicine and Health, Örebro University, Örebro, Sweden; b Faculty of Health, Science and Technology, Department of Health Sciences, Karlstad University, Karlstad, Sweden; c Faculty of Arts and Social Sciences, Department of Social and Psychological Studies, Karlstad University, Karlstad, Sweden; d Faculty of Arts and Social Sciences, Center for Service Research, Karlstad University, Karlstad, Sweden

**Keywords:** Family violence, intimate partner violence, health care professionals, qualitative methods

## Abstract

**Purpose:**

This study aimed to explore child health care nurses’ clinical experiences from encounters with mothers exposed to intimate partner violence (IPV), as little research has explored this topic.

**Method:**

Nine child health care nurses from two Swedish regions were interviewed. The interviews were analysed using thematic analysis.

**Results:**

The narratives depicted the nurses’ strong commitment to, and professional relationship with, the exposed mothers. The experience of working as a nurse and having encountered IPV in clinical practice made the nurses more confident, which impacted their performance and attitude towards this topic. The ability to uphold the professional relationship was threatened by lack of support and interprofessional collaborations.

**Conclusions:**

The professional relationship was central to the encounters, yet could impose an emotional burden on the nurses. While the nurses wanted to improve their knowledge of the process around the mother and child, they were happy to pass the primary responsibility over to other professionals. The findings highlight the challenge in establishing sustainable support for nurses, and building a transparent collaboration process between the health care sector and the social services, serving the well-being and safety of the mother and child.

## Introduction

The detrimental risks of intimate partner violence (IPV) exposure to mothers’ health and wellbeing, as well as to children’s health and development, are well established (World Health Organization, 2017). Although prevalence has been shown to increase in adverse life circumstances, such as poverty and societal upheaval, IPV constitute a major health problem in Europe and in Sweden. In a European survey, 23% of the women had been exposed to sexual and/or physical IPV during their lifetimes (European Union Agency for Fundamental Rights, 2014). In Swedish surveys, lifetime prevalence between 14–28% have been reported (National Centre for Knowledge on Men’s Violence against Women, NCK, 2014; FRA, 2014). Mental health problems, such as depressive disorders, and post-traumatic stress disorder, physical health problems, such as high blood pressure and severe obstetric complications in mothers; neurological deficiencies, language delays, and various mental health symptoms in children are some of the numerous negative consequences of IPV (Bauer et al., 2013; Hahn et al., 2018; Holt et al., 2008; Udo et al., 2016). The negative effects on children, especially when IPV is combined with other adverse childhood experiences, which is frequently the case, may persist lifelong, with significantly increased levels of common physical diseases and mortality (Bellis et al., 2015). IPV is a risk factor for insecure attachment and dysfunctional maternal behaviour associated with emotional dysregulation in children (Sancho-Rossignol et al., 2018). Furthermore, IPV is associated with health problems in the mother–infant dyad both in the short and long term (Howell et al., 2016); thus, it is a critical issue warranting attention from both child and maternal health care professionals.

The high health risks for both mothers and children are of global concern and the fact that few women voluntarily report their suffering, have made IPV screening a highlighted issue within health care. The American Academy of Paediatrics initiated advocacy for IPV screening in paediatric health care settings as early as 1998 (Prakash et al., 2019). However, general screening within primary child health care is under debate and national recommendations and practices concerning IPV screening vary globally. The World Health Organization (2013) recommends screening for IPV during pregnancy, but not postpartum. The arguments against routine postpartum questions about IPV underscore the lack of evidence of beneficial effects for the mothers, as well as concerns among child health care nurses regarding stigmatizing abused women or increasing the risks for these women (Elliot et al., 2002; Klevens et al., 2012). Some health care providers express a great deal of personal ambivalence and anxiety about screening, and several myths about the negative consequences of IPV screening have been described. These include concerns that asking about IPV will damage the provider-patient relationship, and a belief that the recognition of IPV by a health care provider will not change anything, as it cannot prevent the violence (Davies et al., 2015). In a Canadian trial in multiple health care settings the results did not provide sufficient evidence to support IPV screening in health care settings (MacMillan et al., 2009).

Nevertheless, general screening has in Swedish and American studies been described as a preventive intervention to counter isolation and silence about IPV (Anderzen-Carlsson et al., 2018; Davies et al., 2015), and to demonstrate that child health care is an arena where sensitive topics with negative health effects on children can be discussed (Anderzen-Carlsson et al., 2018). When Swedish mothers were asked about their opinion of IPV screening within child health care, they were very positive and expressed that IPV screening is necessary for the sake of the children (Almqvist et al., 2018b).

Despite the ongoing international debate, postpartum screening for IPV within child health care has been implemented in some countries, such as Australia and some regions of Sweden (Almqvist et al., 2018a; Taft et al., 2015). However, we argue that it is important to follow up on such implementations, which is in line with MacMillan et al. (2009), who state that evaluation of services for women after identification of IPV should be a priority. An Australian two-year follow-up study of an implementation of routines working with IPV in the health care sector identified that national guidelines for routine questions about IPV seem to require a sustainable complementary system of support and training for child health care nurses, as well as financial resources. In the absence of such a system, the established routines related to identification and care risk deterioration (Hooker et al., 2016).

Although there is previous research focussing on screening for IPV within the health care sector in general, and within maternal and child health care specifically, studies investigating health care nurses’ experiences of recognizing women exposed to IPV are sparse (Garcia-Moreno et al., 2015). Even more specific, research focusing on exploring child health care nurses’ experiences of encountering IPV exposed mothers is limited, yet needed as a means to evaluate the process following identification of IPV and for understanding of how to best support a sustainable process for the ones concerned. Nurses’ experiences when coming to know that a mother and child are exposed to IPV may increase or decrease their willingness to inquire about IPV in future professional contacts.

Therefore, this study aimed to explore child health care nurses’ clinical experiences of encountering mothers exposed to IPV.

## Methods

### Study design

This study used an exploratory, descriptive design using qualitative methods. Qualitative methods are regarded as appropriate when the aim of a study is to understand an area that has been sparsely described and/or when the aim is about understanding a phenomena (Morse & Richards, 2002), both were relevant for the present study. Data was collected using individual semi-structured interviews and were analysed using thematic analysis (Braun & Clarke, 2006). This method aims to identify, analyse, and report patterns within the data.

### Setting and participants

Child health care nurses (CHCNs) at all 59 child health clinics in two regions in Sweden were invited to participate in the study. The clinics varied in size, with the number of newborns ranging from 35 to 362 per year, and the total number of enrolled children (newborn to five-year-olds) ranging from approximately 200 to over 2000. Organization managers and child health care psychologists shared information related to the study with CHCNs in routine meetings, as well as in informal meetings. The inclusion criteria were as follows: a) having experience of recognizing at least one mother exposed to IPV on duty, and b) having contact with the exposed mother for some time following the identification of IPV. Interested CHCNs who met the inclusion criteria were asked to contact the researchers or share their telephone number with the organization managers or child health care psychologists for the researchers to contact them for more information. The sampling strategy was one of convenience; all eligible CHCNs who were interested in participating in the study were included.

Nine CHCNs from seven different child health clinics volunteered and consented to participate in the study. All participants were women, as representative of the population. Their work experience ranged from a couple of years to several decades, and they came from a variety of different child health clinics in terms of catchment area, location, and organizational structure. The CHCNs also had varied experiences of meeting mothers subjected to IPV, ranging from one experience to several experiences, positive and negative experiences, and recent and older experiences.

The seven clinics represented the demographics of the two regions, being situated in both major cities and rural areas, and low- and high-income neighbourhoods. The mothers attending the child health clinics were predominantly of Swedish origin (86%), with 4% having European and 10% non-European origin.

At the time of data collection, there was an ongoing focus on IPV in the two regions, and during the two years preceding data collection, interested CHCNs had participated in an intervention study on the topic. Following the intervention, the management of the child health clinics decided to implement a routine of asking every mother in the clinic about IPV when their child was approximately two months old, using a structured questionnaire called ‘Violence in the Family’ (Hultmann et al., 2014). An internal follow-up study, conducted over a three-month period a year after the implementation (Almqvist et al., 2018a) showed that the new routine was adhered to in more than 95% of the visits in one of the regions. The implementation process was delayed in the other region, and at the time of the follow-up study, IPV screening was performed in 64% of the visits in the child health clinics which had implemented the routine.

### Data collection

Semi-structured interviews were conducted with the CHCNs. The focus of the interviews was to explore CHCNs’ clinical experiences of encountering mothers exposed to IPV. Two researchers individually conducted the interviews (the second and the last authors) during the latter half of 2017. The second author is qualified with a PhD in psychology with research experience using qualitative methods, and the last author is a licenced psychologist, licenced psychotherapist, and professor in psychology. Neither of the two had any prior clinical or professional relationship with the interviewees. The duration of the interviews ranged from 45 to 90 min. All interviews were recorded using a digital recorder with the permission of the participants, and were transcribed verbatim.

This study was approved by the Regional Ethical Review Board in Uppsala, Sweden (registration number 2017/300).

### Data analysis

A thematic analysis of the interview data was undertaken, informed by Braun and Clarke (2006) method. The transcripts were independently read several times by the authors, for their interpretation of the CHCNs’ narratives regarding their experiences, including feelings and thoughts from having met a mother who was or had been subjected to IPV (familiarizing with the data). Second, the authors made notes on the content (generating initial codes). These were later collated into potential themes, which were discussed between the authors until a consensus regarding the themes was reached. The themes were then checked against the data to ensure accuracy, and thereafter defined and labelled with a name. Finally, a report was prepared based on extracts from the data.

## Results

The analysis revealed five themes representing different aspects of the CHCNs’ clinical experiences from encountering women who had been subjected to IPV: *Encountering IPV implies a professional commitment and burden, Knowledge and experience (of IPV) makes the CHCN more confident, Not always a given to take immediate action and initiate collaboration (with social services), Interprofessional collaboration is a prerequisite for dealing with IPV*, and *Disappointment with available resources and outcomes for the various family members.*

Below, the themes are organized as headings under which the content of each theme is first summarized and then described in more detail, with the help of verbatim narratives from the individual participants to allow for confirmability of the findings. Finally, a thematic summary will be presented illustrating a latent common thread that runs through the themes; that is, the importance of the establishment and maintenance of a professional relationship.

### Encountering IPV implies a professional commitment and burden

The CHCNs were committed to their work, which included identifying and taking action if they recognised a woman subjected to IPV. Despite their commitment, identifying and working with IPV awakened various feelings in the CHCNs, which needed to be addressed. Encountering IPV constituted an emotional burden, and it could also increase workload in an already strained schedule at the child health clinic. When identifying IPV, the CHCNs often took on the responsibility of keeping in contact and maintaining a professional relationship with the mother, even if she was also referred to another professional or authority for support. The CHCNs wished for an improved everyday life for the family involved, and when this was the case, their narrative had a happy ending, illustrating a successful process.

As mentioned in the summary, encountering women subjected to IPV was emotional for the CHCN: “*What happened to me then, I was not prepared for the emotions it would awake and I went out jogging when I got home. It was difficult, because one gets so affected with the awareness of peoples’ lives.”* (CHCN #3)

It could be difficult for the CHCNs to face the adverse circumstances of some women’s lives, and to question whether their own professional contribution to help the woman was enough. In addition, this took time from other tasks at the CHCNs and caused stress. Another burden for the CHCNs was when they could find no place where they could refer the mother whom they had identified as being subjected to IPV. In such cases, CHCNs often felt exposed in their professional role.

Well, I’ve been thinking, I would like clearer guidelines; we do this and that. We contact these people, and they take on the responsibility [for the woman], because now it felt like it is all back in my lap, and I’m not trained as a psychologist or social worker. I’m no expert on these types of things. No, I think, when I have a question, I want a clear answer. I want to know where to refer [the woman], because my knowledge isn’t enough in this situation. (CHCN #2)

Some of the CHCNs were worried about their own safety and being subjected to violence from the mother’s perpetrator. They wished for an alarm to be installed or to have locked doors at the child health clinic. In some cases, this fear was addressed by always having at least two professionals in the room when the perpetrator was present.

Despite the emotional hardships in encountering women exposed to IPV, the CHCNs often kept in close contact with the mother, before referring her to a counsellor or to social services. The CHCNs offered the mother extra follow-up visits for the infant, to check how things were going for the mother and child, and to let the mother know that they were there for them. The CHCNs also maintained a professional relationship with the mother later in the process when the mother had been referred for additional support.

Well, one always meets with them, and asks how they are doing, yet after having referred them, you don’t want to have the main responsibility. Now, you can really feel, ‘Now I can let go of this, now it’s someone else that’s in charge’. But still, every time one meets with them [the mother and child] it is natural to ask how things are going. But, then, the major burden is for someone else to carry on their shoulders. (CHCN #7)

The CHCNs felt that this close contact with the mother was important for the mother and gave the CHCN a sense of control. When a mother was referred to another child health clinic, due to moving away from the perpetrator, the CHCNs sometimes felt a sense of loss.

### Knowledge and experience (of IPV) makes the CHCN more confident

The general experience of working as a CHCN, and of having encountered women exposed to IPV made the CHCNs more confident, which had an impact on their performance and attitude towards dealing professionally with IPV. Nevertheless, finding a private zone for talks with the mothers, when the partner did not allow the woman to be alone with the CHCN, could still be a challenge. Over time, the CHCNs, however, developed strategies to work around the partner’s control.

When recalling early experiences of women subjected to IPV, the CHCNs expressed not knowing what to do at the time, which caused them to doubt their professional manner.

I have had a woman confessing, during a ‘private mother-talk’, that she was subjected to IPV. At that time, it became really burdensome, because at that time I didn’t even have, well, we had not been given any education about talking about it. I hardly knew *…* I had to run to the midwives, to ask for a telephone number [for the woman to call]. (CHCN #3)

With more experience of encountering women subjected to IPV, and with tailored education and better guidelines, the CHCNs felt more secure when inquiring about IPV. The CHCNs trusted their intuition when they sensed that something felt off, and were convinced that asking about IPV in a straightforward fashion was the right way to address the issue. Often, it resulted in honest answers, or the mother became aware that the CHCN was there to listen to her, if she decided to talk in the future: “*So I asked her once [about IPV], and she said, ´No, it has been a long time since he has beaten me, not nowadays´, but then, after a few visits, she brought the subject up herself and told me, ´It’s not true, he still beats me’”*. (CHCN #5)

With experience, they were also more likely to speak clearly to the mothers about IPV, and to make the mothers aware that they were actually exposed to IPV when a mother revealed psychological or physical abuse from their partner, which was not acceptable. They also talked about how they confirmed the mothers’ experiences and supported them when they had already decided to leave the perpetrator.

This woman, who didn’t want to tell me more [refers to an earlier part of the interview], well, what I could do was to validate her; she was right, violence isn’t okay, and I said to her that it was the right decision for her to leave him. (CHCN #3)

### Not always a given to take immediate action and initiate collaboration (with social services)

The CHCNs rarely made immediate contact with the social services when they realised that a mother was being subjected to IPV. Instead, they calculated the severity of the abuse and the risk for the mother and her child. If it was deemed that the mother and child were at no acute risk, the CHCNs often regarded it better to establish a relationship with the mother with the goal of obtaining her consent to inform the social services. The CHCNs then actively worked to make the mother aware of the situation, offered advice to her to initiate support herself, or arranged for support within the child health care organization. However, if the child was regarded as suffering as a result from the IPV, the nurse did not hesitate to report this to the social services.

The CHCNs described being aware that there was always a potential risk for the health of the child when the mother was subjected to IPV. Thus, they wanted to offer immediate help to the mothers, often within their own organization. However, frequently the CHCNs did not immediately report the abuse to the social services, as they believed that it was important to obtain the mother’s consent first, or at least inform the mother when reporting to the social services. The participants revealed several reasons for this. A common reason was the fear of jeopardizing the professional relationship, although this fear tended to subside as the CHCNs gained more experience.

The case was that there were other CHCNs to turn to, if they lost confidence in me, but today I’m more focused, I cannot back-off reporting, just to make them keep on liking me. I guess that this realisation has come with experience. (CHCN #5)

Other reasons were the fear of hurting the family and wanting more information about the situation before acting. Support from CHCNs or other professionals within the child health care organization could lead to awareness in mothers that they were subjected to IPV, which in turn could lead them to end their abusive relationships; this was a reason for the CHCNs to postpone reporting to the social services. The mothers were often given information about alternative support agents, and at times were left to decide what they wanted to do. However, when the CHCNs identified that the child was at risk, they described the importance of immediately reporting this to the social services. In such cases, they saw no alternatives.

It was because of his [a child] behaviour towards the mother that we filed a report about the children’s situation to the social services, because they were so affected by this destructive relation. They [the children] had started to show symptoms of the parents’ pathological relation, and thus we had to file a report. (CHCN #6)

Often, but not always, the parents were informed in time by the CHCNs about such a report. In one case, a mother had later on asked the CHCN to withdraw the report she had filed with the social services, which is not possible according to Swedish legislation.

### Interprofessional collaboration is a prerequisite for dealing with IPV

Interprofessional collaboration was described by the CHCNs as necessary to help the family members involved in IPV. It included professionals from their own organizations, as well as from other organizations. However, the collaboration was not described to always take place on equal terms, as the CHCNs often felt that they were not informed about actions that were taken by, for example, the social services. Interprofessional collaboration within their own organization, such as collegial support and counselling for the professional performance and well-being of the CHCN was described as being of great importance.

The CHCNs emphasized that they needed support to be able to support mothers subjected to IPV. This need for support was two-fold: first, to manage the emotions that were awakened by listening to women in a vulnerable situation. Second, to get advice on the most appropriate course of action. In these situations, colleagues at the CHC could be the first level of support.

Yes, we talk a lot to each other, us CHCNs, but we also receive valuable support from NN [an external multidisciplinary team], because there are social workers and psychologists, and as we are localised in the same building, we can sort of think aloud together, yes that’s really facilitating. (CHCN #4)

It was also common to have regular consultations with child health care psychologists. Besides it being a prerequisite for their own wellbeing, the CHCNs also highlighted the importance of interprofessional collaboration for the women and children involved. The CHCNs expressed a desire to be able to refer the women subjected to IPV for further support, and at times, for specific support for the children. This referral was often to a child health care psychologist or social worker within the social services.

The CHCN observed that interprofessional collaboration was facilitated when they were working physically close to other professionals who had offices close by. This meant that it was easy to just pop-in to ask for advice, and the professional knowledge of each individual facilitated fruitful collaboration. One organizational structure mentioned as a valuable resource was the ‘Family Centre`, where professionals from both the child health clinic and the social services had their offices in the same building and offered joint family-activities. Here, the CHCN could talk informally with professionals from the social services and ask for their advice. The various professionals involved in the Family Centre were described as complementing each other’s competences at the various steps in the process of encountering families where the woman was subjected to IPV: *“ … but this case was very special and because of that it was very comforting being situated at a Family Centre, because all of us in this house were involved later on in the process*.” (CHCN #9). Interprofessional collaboration in the Family Centre was at its best when various professionals could observe the families and talk to them together in a more relaxed manner, as compared to the CHCN merely referring the families to the social services.

At times, the interprofessional collaboration worked fine and the CHCN participated in meetings with the social services and parents despite not having their offices nearby or collaborating in Family Centres. Sometimes, the mother, with whom the CHCN had an ongoing contact, initiated and facilitated such meetings. However, a lack of mutual collaboration with the social services, after having reported a child at risk due to IPV, was also described. *“Well … the social services don’t reveal anything to us. They hardly said anything although I was the one filing the report, it is really terrible, because we are taking care of the same children.”* (CHCN #8). Not having any information about what actions the social services had taken was regarded as a hindrance for the CHCNs in their ongoing work with the family. If the CHCNs were knowledgeable about the family situation and what actions were taken on the behalf of the social services, it was easier for them to talk to the parents about the observed problems and the situation at home.

Generally, the CHCNs believed that if the social services were more responsive to them after they had filed a report, as a group, they would be more likely to report when a child is at risk. Some CHCNs believed that the only consequence of filing a report was having the parents become angry with the CHCN, and perhaps ending the professional relationship with her, thereby taking away her ability to help.

Then we [the CHCNs] see the point with reporting [if having feedback from the social services], because we all know that CHCNs and midwives are not good at reporting to the social services. It could be because we never have any feedback, or we believe that the social services don’t take any action, which I believe they actually do, but we never hear anything about it. In most cases, the only thing that actually happens is that the parents get upset with us, which affects our relationship with them, and in those cases, they don’t reveal anything to us anymore. (CHCN # 1)

In addition to the social services, the CHCNs also talked about the positive experiences of interprofessional collaboration with the police, either when witnessing violence or when being worried about a mother’s situation at home. Furthermore, collaboration with non-governmental organizations and local state churches was also mentioned as beneficial in the work with mothers subjected to IPV and their children.

### Disappointment with available resources and outcomes for the various family members

The CHCNs missed specific support for children who had witnessed IPV, and thus were victims of abuse, as well as support for the perpetrating fathers. However, the CHCNs were more concerned that the mothers did not receive, in the CHCNs opinion, sufficient support from child health care organizations and the social services.

The CHCNs highlighted that while the new routine of asking all new mothers about their experience of violence had been implemented, a routine of the follow-up of positive answers was lacking.

What is lacking are clear guidelines and instructions on what to do with these women. The way it is now does not work well. It gives me a sense of a process not being finalized; they have not been thinking of the whole line. We [the CHCNs] are instructed to start something, yet no preparations of how to take care of the results are in place. (CHCN #2)

The CHCNs were also frustrated and disappointed with the difficulties of referring mothers and children.

These cases, when we don’t receive any support, it makes you frustrated, or feel powerless, ‘What am I supposed to do next?’ … … when bringing up the topic … … despite this, sometimes nothing happens … … so that’s what I wish for, a straightforward way to go. I believe that this would save a lot of effort. (CHCN #8)

The psychologists at some child health clinics did not want to take on consultations with children, as they did not regard themselves as having the required knowledge and skills. Sometimes, child and adolescent psychiatry also denied referrals from the child health clinics. At other times, the waiting list was long, which was frustrating for the CHCNs.

I believe that the entire society must look at this in another way; we must protect the children, and not only think from the perspective of adults’ needs. We need to have an open dialogue with the young children who have witnessed violence, to assure them, so that they don’t blame themselves for what happened. (CHCN #1)

In line with this, the CHCNs experienced that the social services and the Family Court did not always have the child’s best interests in mind. Sometimes, no investigation of the families’ situation was initiated, although the CHCNs had referred mothers to them or filed a formal report to the social services. At times, the social services or the Family Court claimed that the child should live part-time with both parents, if the mother had left the father after having been subjected to IPV. Similarly, the CHCNs had experienced that women’s shelters were often full, and the mother and children were instead appointed to a hotel or a hostel, an environment unsuitable for family life. Additionally, sometimes, mothers did not receive any financial support from the social services, which forced them to move back to the perpetrator. These cases were a source of great frustration for them.

Another unwanted outcome, described by the CHCNs, was when mothers declined the help offered by the child health clinics or the social services. One reason for the latter was that mothers experienced stigma related to having contact with the social services. The consequence was that the mother ended up being without any support other than the support from the CHCN, which according to the CHCNs was an unfavourable and disappointing outcome of the initial intervention.

The CHCNs were not only disappointed with a lack of available resources and the outcome of their referrals, but they also reported a lack of support for the perpetrators, that is, the fathers. On a more general level, they recognized that the work at the child health clinic focused more on the well-being of the child and the mother, and felt that the father often became invisible in this setting. One thought was that it would be a good idea to offer preventive individual talks with all fathers, similar to the private talks with all mothers, which are part of the national guidelines for child health care.

I believe that if you are suffering from mental health issues to some degree, you are more likely to be violent. In a gender equal society, like ours, I think we should be more aware of the health status of fathers as well. Why don’t we offer individual ‘father-talks’? Then, his frustration might be released here, and he can be offered tailored support, instead of beating up his wife. (CHCN #1)

### Thematic summary

The identified themes are mutually exclusive but interrelated; a common thread that runs through them is the importance of the establishment and maintenance of a professional relationship between the CHCN and the mother. The mothers seldom revealed being subjected to IPV on their first meeting with the CHCN. It was after repeated meetings with the mother, which is common in the child health clinics during an infant’s first year, and after a professional relationship had been established, that a suspicion regarding IPV was awakened or revealed. In such cases, the relationship typically became more emotionally loaded, and the CHCN intensified the contact, to observe the health of the mother and infant, and to get more information about their situation. The aim of the intensified contact was for the CHCN to decide whether she had to take action (and perhaps putting the relationship at risk), or whether, based on the relationship and the mother’s consent, she could contact the social services or refer the mother for further help and support within the child health care organization. Regardless of which option was chosen, the CHCN tried to maintain a professional relationship with the mother to stay updated with the developments in the situation. In order to manage the strain of the relationship and offer the best possible support to the mothers, the CHCNs themselves require support and collaboration with colleagues and social workers. In the absence of such support, or in cases where CHCNs who have invested time and effort in their commitment to help the mother, and indirectly the child, feel excluded from the process. They may also experience negative emotions, such as worry and frustration.

## Discussion

The aim of this study was to explore CHCNs’ experiences of clinical encounters with mothers exposed to IPV in their child health clinic practice. The results show that the general experience of having encountered IPV made the CHCN more confident, which had an impact on the their professional performance and attitude towards IPV. The CHCNs’ narratives provide a picture of a strong commitment and a professional relationship, as well as an array of negative experiences, most often related to lack of support and interprofessional collaboration, threatening their ability to uphold the professional relationship.

Some of the findings related to encountering mothers subjected to IPV are not unique, neither in the Swedish setting, nor for CHCNs. For example, our results regarding commitment and feelings of confidence based on the experience of working as a CHCN, and of having encountered IPV in practice, are similar to the findings reported by Henriksen et al. (2017) and Hegarty et al. (2020), that is, professionals’ personal interest in IPV facilitated asking about IPV. Contrary to a study by Sundborg et al. (2015), who found that district nurses, who feared reacting emotionally and thus became too involved, avoided asking women about IPV, the CHCNs in the present study did not fear this involvement. Indeed, the CHCNs’ emotional involvement was more a result of their professional commitment and a strong driver to help mothers and children subjected to IPV.

Despite their willingness to approach IPV it could still be perceived as a burden, as the CHCNs felt that responding to IPV took a lot of time in an already strained schedule. This is in line with previous studies (Henriksen et al., 2017; Sundborg et al., 2015) and has also been determined as a barrier for approaching the topic (Sundborg et al., 2015; Yeung et al., 2012). Time restraints as well as having to prioritize time between tasks that are considered equally important (e.g., other patients), are known to cause moral distress (Kälvemark et al., 2004), which could be a contributing factor to the CHCNs’ expressed burden.

Taken together, some of the main stressors among CHCNs were lack of support from their own organization; lack of guidelines and functioning routines, where the CHCN could find both emotional and professional support, lack of information from social services concerning the fate of the mothers and children, as well as fear of their own security. These results are similar to previous findings reporting these as barriers to asking women about IPV (Henriksen et al., 2017; Sundborg et al., 2015; Williams et al., 2017; Yeung et al., 2012). Moral distress is an important aspect to consider for health care professionals, and can be augmented by lack of supporting structures where health care professionals can give voice to dilemmas (Kälvemark et al., 2004). In the present study, CHCNs called upon support related to their emotional needs as well as the support and resources for mothers and children subjected to IPV. A supportive environment could include collegial support and professional supervision (Sundborg et al., 2015), measures identified as important also in the current findings. In addition, collaborating with, and being supported by a team (internal or outside the organization) were identified, in a meta-synthesis, as having a positive impact on health care professionals’ readiness to discuss IPV (Hegarty et al., 2020), which is also highlighted in the present findings.

Davies et al. (2015) described health care providers’ reluctance to screen for IPV as caused by several myths, including the risk of damaging the provider–patient relationship. The CHCNs’ descriptions in this study add to this knowledge, as they indicated that the fear of damaging the relationship was an issue for the CHCN even after recognition of exposure to IPV. Nursing theorists have argued that the relationship between nurse and patient is fundamental for nursing (e.g., Hildegard Peplau and Joyce Travelbee; in Pokorny, 2014), an attitude which might have implicitly influenced the attitudes among the CHCNs in the present study. Hegarty et al. (2020), found that health care professionals view their professional relationship as the underpinning factor for their readiness to approach IPV. They also highlighted the importance of continuity of care in facilitating an effective response to IPV. Thus, the importance of the relationship might not be unique to nurses. However, a long-lasting professional relationship could also be a barrier, as some patients might not want to disclose IPV if they are too familiar with the professionals (Yeung et al., 2012), which might be important to keep in mind.

In the present setting, the professional relationship should not only be limited to the mother subjected to IPV, as it also involves at least one child, and thus the CHCNs must pay attention to the child as well. The CHCNs in the current study seemed to focus more on the wellbeing of the mother, and on creating an alliance with her, which could be interpreted as them prioritizing this relationship over the child’s right to protection; why it was not always a given to take immediate action and report the abuse. This could be a potential dilemma, as the CHCNs, by this attitude, set aside their legal obligation to promptly report any suspicion of child maltreatment to the social services. This too could possibly cause moral stress; according to Kälvemark et al. (2004), deliberate rule breaking can cause moral distress in health care professionals, as this creates a conflict between what they know is right (e.g., file a report to the social services) and what they think is best for the individual concerned (e.g., observe the situation and wait for the mother’s approval for contacting the social services). According to our findings, the CHCNs did not regard this as a problem, but clearly stated that when they identified that the child’s health was at risk, they did not hesitate to report this to the social services. Until then, they justified their actions as the right course of action according to the premises given to them. This attitude is similar to that reported by Hegarty et al. (2020), who identified that healthcare professionals adopted an advocacy approach when encountering women subjected to IPV, including working in alliance with them. Although understandable from the perspective of safeguarding the relationship with the mother, the CHCNs’ descriptions of waiting to report the abuse until the mother consents to it, is not in accordance with the Swedish legislation. Professionals who work with children are obliged to immediately report IPV as well as child abuse to the social services, as soon as they suspect it (Social Services Act, 2001).

The CHCNs found it difficult if the relationship with the mother ended without the CHCNs’ interventions being completed successfully, such as if the mother moved from their catchment area, or when the CHCN was disappointed with the available resources and services offered by, for example, social services. According to Peplau (Pokorny, 2014), the last step in a patient–nurse relationship is the resolution phase, where the client no longer needs professional services and gives up dependent behaviour, which is when the therapeutic relationship comes to an end. This could sometimes be difficult for both parties; as psychological dependence could persist. The findings reveal that the relationship coming to an end was at times difficult for the CHCNs, having committed to the professional relationship. The findings concerning the importance of maintaining the relationship and feeling content with one’s work coming to an end could also be understood in light of the Swedish organization of child health care, where the CHCN’s role is central. The Swedish child health care is free of charge and run by CHCNs, ruled by a national programme, following 99% of all children aged 0–6 years and their parents (Wettergren et al., 2016). Thus, the CHCNs follow the families for many years, especially if the mother has more than one child. The visits are most frequent during the first year of the child’s life, when the CHCN monitors the growth, development, and well-being of the child, as well as the health and social situation of the parents.

Although the CHCNs in the present study stated that they wanted to continue their professional relationship with the mother, they were also clear that they wanted to refer the mother for further support or treatment and to transfer the responsibility regarding the follow-up of the IPV. The importance of referring women to other professionals has also been described in previous studies (Alhalal, 2020; Yeung et al., 2012). A somewhat new finding in this study is that the CHCNs wished to collaborate and be informed by the professional to which the woman was referred, such as the social services. Perhaps, this can be understood in the context of nursing, where collaboration is common. One concept analysis of collaboration in nursing defined collaboration as “an intraprofessional or interprofessional process by which nurses come together and form a team to solve a patient care or healthcare system problem with members of the team respectfully sharing knowledge and resources” (Emich, 2018, p. 569). This notion is probably why the CHCNs valued and argued for interprofessional collaboration, aimed at focusing on the best option for the mother, child, and family, and why they were disappointed when they were left with no information from the social services. It can also serve as an illustration of the different professionals involved, having conflicting values related to their work (cf. Kälvemark et al., 2004), where the social workers adhere to secrecy to a higher degree than the CHCNs, which in turn could result in distress for the CHCNs, as they have no say in the process. However, in line with the current study, previous research has identified that joint actions, such as working with the social services, make nurses more likely to address IPV (Stenberg et al., 2017). Thus, for the well-being of children, it is important to further develop interprofessional collaboration among authorities.

The Family Centre was described as a beneficial organization for CHCNs working with mothers exposed to IPV. At a Family Centre, the social service and the child health clinic are allocated together with an open nursery, offering an opportunity for professionals from different organizations to collaborate on preventive and health-promoting activities (Bing, 2012). When working in common facilities, the problem of lack of information from the social services to the CHCNs seemed to vanish. Interestingly, legislation concerning secrecy and information between organizations remained.

Being allocated together with other professionals also increased CHCNs’ sense of security, and decreased their fear for their own safety. Safe facilities are required if CHCNs are confronting abusive men by supporting mothers exposed to IPV. Today, many CHCNs work alone in their offices and when visiting families in their homes. Hooker et al. (2016) have shown the need for a sustainable system for the support and training of CHCNs for screening routines for IPV in child health clinics to be sustainable. The present study indicates that several aspects of the CHCNs’ workplace and organization need to be altered to minimize the risks of CHCNs being exposed to negative consequences. Some scholars have suggested that IPV should be a part of the nursing curriculum (Alhalal, 2020), and that educating professionals on this topic is needed (Williams et al., 2017). Others have indicated that formal education is insufficient (Sundborg et al., 2018; Yeung et al., 2012); in order to feel confident in talking about IPV, health care staff need training and experience (Yeung et al., 2012) as well as continuous supervision and support (Sundborg et al., 2018).

Taken together, the findings indicate that it is important not only to decide whether to start screening for IPV, but also to ensure that the professionals who are screening have the best resources, such as an adequate level of education and support, and clear guidelines on the process (Alhalal, 2020).

### Strengths and limitations

One major strength of this study is that it focused specifically on CHCNs encountering mothers who were being subjected to IPV. This focus is sparse in extant literature. The study contributes to the current knowledge on the CHCNs' professional role and the importance to find ways to establish sustainable support for CHCNs who may encounter mothers who are subjected to IPV. A supportive environment can ease CHCNs’ emotional burden and contribute to better care for mothers and children concerned. To establish transparent interprofessional collaboration is also of importance. A possible limitation is that the sample size in this study can be regarded as small. Nevertheless, in qualitative studies, the sample size does not determine the quality or credibility of the data (Malterud et al., 2016).

## Conclusion and clinical implications

The findings indicate that the relationship between the Swedish CHCNs and mothers is central to their encounters. Although it is a professional relationship, the CHCNs invest commitment in their work, and in particular with the mothers exposed to IPV, which imposes a professional and emotional burden on them. Moral stress may arise when the mother is not willing to accept help despite putting her child to risk. The CHCNs want to be further involved and stay informed about the process around the mother and child, yet are happy to pass the primary responsibility over to other professionals within their organization or to the social services. They also wish to be better equipped to deal with these situations, and with experience and support, they become safer and more secure with their tasks.

Based on this, one clinical challenge in the Swedish context is to find ways to establish sustainable support for all health care professionals who may encounter mothers who are subjected to IPV. A supportive environment, including collegial support and professional supervision can ease CHCNs’ emotional burden. Another challenge is to establish interprofessional collaboration between the health care sector and the social services, which serves the well-being and safety of both the mother and child. A working interprofessional collaboration will also help the CHCNs feel involved and competent in their further encounters with the affected mothers, and increase their willingness to report to the social services when they identify a child at risk due to the mother being subjected to IPV.

The transferability of the results rests with the reader (Lincoln & Guba, 1985). However, as some of the results are similar to previous results from encountering IPV in other health care settings, it is likely that the experiences identified in this study could be transferred to other Western contexts, including the need for education and support for the CHCNs. In the future, it would be interesting to design an intervention study to implement the health care readiness to change model (CATCH), based on Hegarty et al.’s (2020) meta-synthesis, to increase CHCNs readiness to deal with IPV. It would also be important to investigate the moral distress of health care professionals in relation to approaching IPV as a concern.

## Data depository

The data supporting the results are stored at Karlstad University, Karlstad, Sweden.
